# microRNAs associated with the pathogenesis and their role in regulating various signaling pathways during *Mycobacterium tuberculosis* infection

**DOI:** 10.3389/fcimb.2022.1009901

**Published:** 2022-10-27

**Authors:** Kusuma Sai Davuluri, Devendra S. Chauhan

**Affiliations:** Department of Microbiology and Molecular Biology, Indian Council of Medical Research-National JALMA Institute for Leprosy and Other Mycobacterial Diseases, Agra, India

**Keywords:** miRNA, tuberculosis, pathogenesis, signaling pathways, anti-TB treatment

## Abstract

Despite more than a decade of active study, tuberculosis (TB) remains a serious health concern across the world, and it is still the biggest cause of mortality in the human population. Pathogenic bacteria recognize host-induced responses and adapt to those hostile circumstances. This high level of adaptability necessitates a strong regulation of bacterial metabolic characteristics. Furthermore, the immune reponse of the host virulence factors such as host invasion, colonization, and survival must be properly coordinated by the pathogen. This can only be accomplished by close synchronization of gene expression. Understanding the molecular characteristics of mycobacterial pathogenesis in order to discover therapies that prevent or resolve illness relies on the bacterial capacity to adjust its metabolism and replication in response to various environmental cues as necessary. An extensive literature details the transcriptional alterations of host in response to *in vitro* environmental stressors, macrophage infection, and human illness. Various studies have recently revealed the finding of several microRNAs (miRNAs) that are believed to play an important role in the regulatory networks responsible for adaptability and virulence in *Mycobacterium tuberculosis*. We highlighted the growing data on the existence and quantity of several forms of miRNAs in the pathogenesis of *M. tuberculosis*, considered their possible relevance to disease etiology, and discussed how the miRNA-based signaling pathways regulate bacterial virulence factors.

## Introduction

**Figure 1:**
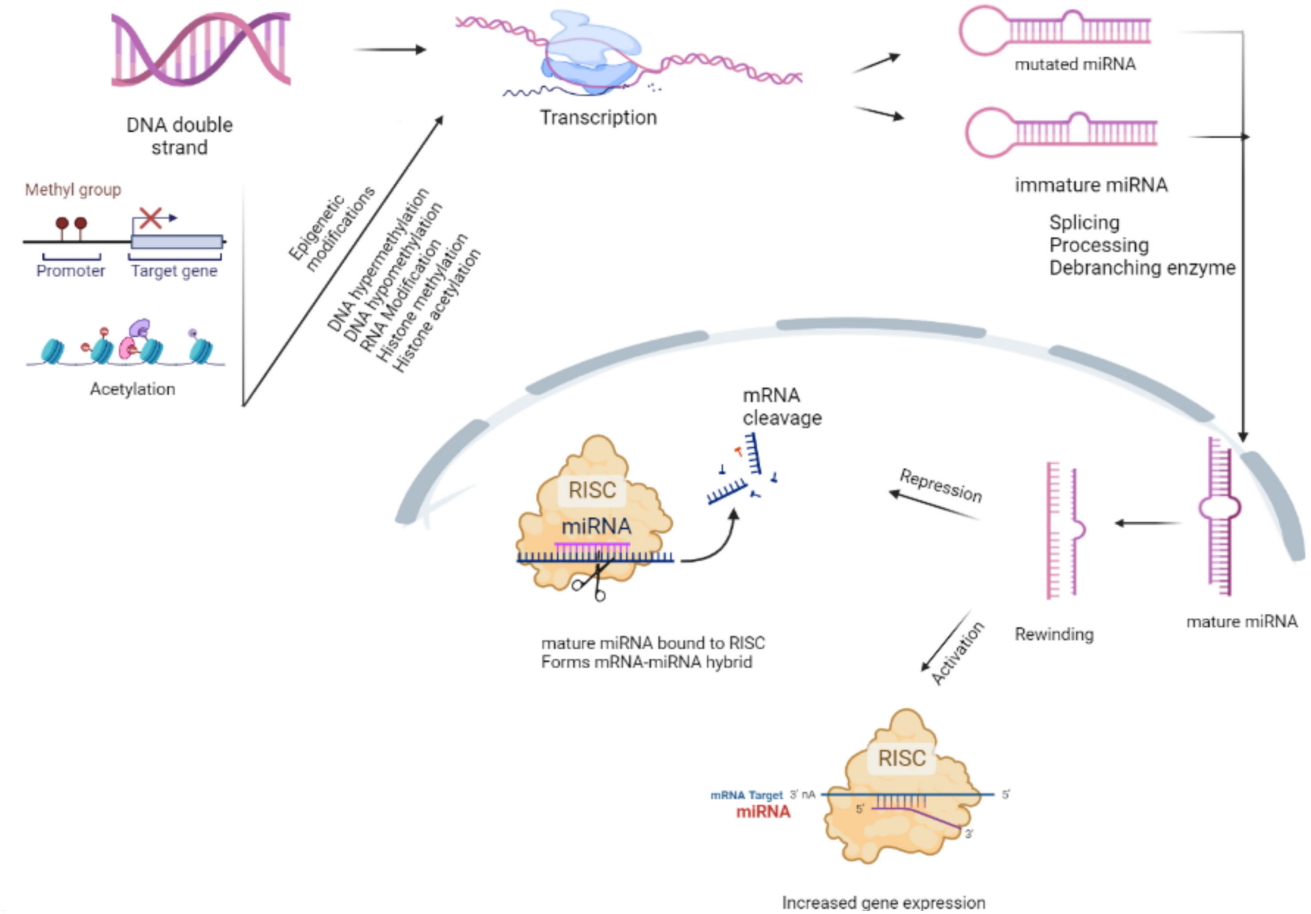
Maturation of microRNA (miRNA). RNA polymerase II or III modifies the primary miRNA into the cap structure through polyadenylation. The Drosha complex crops the miRNA into a hairpin-shaped pre-miRNA during the initial processing of pri-miRNA in the nucleus. Immature miRNA is exported to the cytoplasm by the exportin-5/Ran-GTP complex for Dicer processing. One of the miRNA duplex strands forms miRNA-RISC, which engages on the target mRNA to mediate gene silencing *via* translational repression or mRNA degradation/deadenylation (detailed in **Figure 1**). The epigenetic modifications during the transcription process render the miRNA unable to carry out its normal functions. Messenger ribonucleic acid (mRNA); micro ribonucleic acid (miRNA); deoxyribo nucleic acid (DNA); RNA-induced silencing complex (RISAC).

Non-coding RNAs are single-stranded transcripts that regulate the mRNA (coding gene) expression by degrading them. microRNAs (miRNAs) are small molecules of non-coding RNA that contain 17–25 nucleotides and modulate gene expression (Esteller, 2011). From the past few decades, there is great progress in miRNA research, which is believed to be important in regulating various pathological processes (Aguilar et al., 2019). Over 20 years ago, the first miRNA discovery made a mark in a molecular biology new era. Over 2,000 miRNAs have been identified in humans, and it is thought that they collectively regulate one-third of the genes in the genome. miRNAs have been linked to a variety of human diseases and are being researched for use as clinical diagnostic and therapeutic targets through biogenesis, involving the multitude of mechanisms that inactive miRNA converts into mature miRNA (Taneja and Dutta, 2019). Disruption in the maturation process of miRNA, for example, miR-146a irregular expression and impaired miRNA regulatory mechanisms, leads to neoplasia, ischemic heart disease, neurodegenerative diseases, etc. Yang et al., 2019 (Chen et al., 2019). miRNA formation process occurs in the nucleus by RNA polymerase II. Initially, the primary transcript with hair pin structure that encodes miRNA sequences is regulated by RNA polymerase II transcription factors, epigenetic and histone modifiers. Primary miRNA goes through maturation processes by cropping the loop end of pri-miRNA (pre-miRNA) in the nucleus. Later, the resulting product is exported to the cytoplasm by exportin-5 for further maturation steps. RNase, Dicer crops the loop end one more time resulting in the small RNA duplex (Bogerd et al., 2014). Only six nucleotides that match are required to obtain functional miRNA (Bartel, 2009). Recent research studies reveal the genesis and role of miRNA in regulating several bacterial pathogenesis-associated signaling pathways (Zhang et al., 2017b; Stutz et al., 2018). miRNAs can be reliable in therapeutic settings. These factors became important in screening the diseases with high specificity, sensitivity, and accessibility (Walker and Harland., 2009 Ostrik et al., 2021). Northern blotting, microarray analysis, and quantitative polymerase chain reaction (qPCR) are traditional methods for miRNA detection. To improve the sensitivity and selectivity of miRNA detection, new technology methods always rely on signal amplification strategies, such as nanoparticle-based amplification, isothermal exponential amplification, rolling circle amplification, hybridization chain reaction, and combinations of these (Ye et al., 2019). Our literature review reveals the role of miRNAs as modulators of signaling pathways in tuberculosis (TB). miRNAs act as genetic switches that make them regulators of cellular signaling pathways. We can predict the targets of miRNA easily nowadays through the discovery of high-throughput genomic screening methods. Understanding the role of miRNA in signaling pathways might lead to novel therapies. New kinase inhibitors are being studied to treat many diseases by detailed understanding of the role of miRNA in regulating the kinase cascade pathways. We will indeed be able to create new therapeutical platforms, such as locked genomic technology, for synthesizing and providing efficient RNA-based chemotherapeutic agents. miRNA expression patterns differ in active TB, latent tuberculosis infection (LTBI), and healthy individuals (Sabir et al., 2018). miRNA synthesis mainly influences the action of various immune cells (Chandan et al., 2020). We summarize some of them and discuss their benefits and drawbacks for improving miRNA detection design. Research studies found significant variations in miRNA patterns that help in identifying LTBI and long-term TB infection. Compared to LTBI, active tuberculosis shows upregulation of miR-194-5p, miR-21, miR-29c-3, miR-150-5p, miR-365a-3p, miR-223-3p, miR-451a-5p, miR-44-5p, and miR-144-3p (Wang et al., 2011). *Innate immune response*: miR-146a (IRAK)-1/ (TRAF)-6], miR-9 (NF-κB1), miR-125b (ERK)1 (Zhou et al., 2010), miR-26-5p (KLF4), miR-132-3p [(TLR)]; *Regulation of inflammation:* miR-21-5p (TLR4), miR-146a-5a (TRAF-6), miR-20b-5p (NLRP3), miR-223-3p (NFIA), miR-27b-3p (Bag2), miR-99b-5p [(TNF)-α and TNF receptor superfamily (TNFRSF)-4], miR-125-5p (TNF-α), miR-142-3pN (Wasp), miR-144(IFN-γ and TNF-α), miR-27a (IRAK-4); *Autophagy*: miR-155 (Rheb), miR-27a (Cacna2d3), miR-889 (TWEAK), miR-106a (ULK1, ATG7, ATG16L1) (Yang J et al., 2019), miR-125 (DRAM2), miR-142-3p (ATG16L1) (Yang Y et al., 2019), miR-17 (ATG7), miR-144-3p (ATG4a), miR-20a (ATG7/ATG16L1) Cui et al., 2022a, miR-23a-5p (TLR2/MyD88/NF-κB), miR-26a (KLF4); *Apoptosis:* miR-27a, miR-96 (FOXO3) (Guttilla and White, 2009), miR-20a-5p (JNK)2, miR-27b (Bag2), miR-21 [PI3K/Akt NF-κB], Let-7e (Caspase-3), miR-29a (Caspase-7) (Pattnaik et al., 2022). In this review, we focused on the six main signaling pathways involved in the major pathogenic mechanisms such as autophagy, inflammation, and apoptosis. miRNAs that show strong research evidence of regulation according to the target scan and miRbase software were discussed. Finding out the function of various miRNAs in the regulation of various pathogenic signaling pathways may lead to identifying new therapeutic targets. Inactive mRNA undergoes splicing/processing to convert into mature mRNA. Mature mRNA then transported from nucleus to cytosol where it is translated as shown in **Figure 1**.

## microRNAs in tuberculosis

miRNA expression patterns in patients with active TB were shown to be distinct from those of individuals with LTBI or healthy controls (Fu et al., 2011; Harapan et al., 2013; De Araujo et al., 2019). miRNA synthesis may influence the activation of natural killer cells, macrophages, dendritic cells, and T cells (Xu et al., 2019). To avoid the immune system, *Mycobacterium tuberculosis* may either enhance or inhibit miRNA expression. TNF-α and interferon (IFN)-γ are the host cytokines associated with autophagy during bacterial infection. Myeloid cells triggered by TLR signaling have been demonstrated to be negatively affected by higher levels of miRNA-146a-5p, miR-21-5p, miR-155, miR-199b, and miR132-5p (Kim et al., 2017). The overexpression of miR-27a-5p and miR-33 in *M. tuberculosis*-infected cells inhibits the creation of autophagosomes and the killing of *M. tuberculosis* by macrophages (Liu et al., 2018). *M. tuberculosis*-infected macrophages overexpress miRNAs that target IFN-γ and TNF-α, which suppress the immunological response against *M. tuberculosis* (Chakrabarty et al., 2019). As an additional line of defense against intracellular infections, host miRNAs such as miR-325-3p and miR-20b-5p impact cell death and inflammasome activation (Lou et al., 2017; Fu et al., 2020). Host innate and adaptive immune systems, such as miR-155-5p and let-7f, both have a role in the activation of miRNAs during *M. tuberculosis* infection, which is necessary for the clearance of pathogens (Rothchild et al., 2016). In cohorts that comprised persons with LTBI, active TB, and healthy controls, researchers have studied miRNA expression profiles in serum/plasma or blood cells (Lyu et al., 2019). Compared to LTBI, the expression of five miRNAs was higher in TB patient PBMCs (miR-365a-3p, miR-223-3p, miR-451a-5p, miR-44-5p, and miR-144-3p), with target predictions pointing to a possible role in TB patients’ hematopoiesis. According to another research, active TB patients had an improved expression of miR-194-5p and other miRNAs, including miR-21, miR-29c-3, and miR-150-5p. Upregulation of miR-29a-3p was shown to be a helpful prospective biomarker for qRT-PCR-based differentiation among active TB and LTBI (Zhang et al., 2014; Kanniappan et al., 2017). Individuals infected with HIV and those who were not exhibited similar levels of miRNAs.

It was observed that miR-1246, miR-2110, miR370-3p, miR-28-3p, and miR-193b-5p were overexpressed in active TB, whereas miR-3675-5p was downregulated (Duffy et al., 2018). There was no validity testing done on the patients in the second cohort. Pathological Biomarkers for Tuberculosis Progression and Therapy Response researchers want to find predictive miRNA signatures for LTBI-to-TB progression and anti-TB medication response. According on published data rather than a screening in the lab, these miRNAs were selected. TB patients who received successful TB treatment were shown to have lower levels of other miRNAs than those who did not react to treatment (Lyu et al., 2019). While the concept and therapeutic regimen were the same, the screening method was different in a Chinese study. Compared to miR-148b-3p, miR-92a-3p, and miR-21-5p, miR-125a-5p was elevated in this case (Zhao et al., 2013; Duffy et al., 2018). Due to discrepancies in results, standardization of screening techniques is needed to provide more accurate results. In patients with active TB, LTBI, and isoniazide-treated LTBI, researchers found three miRNAs (let-7a-5p, a small nucleolar RNA miR-196b-5p, and SNORD104) as highly sensitive classifiers to distinguish TB from non-TB group members using insignificant RNA sequencing (RNA-seq) of whole blood (Barry et al., 2018).

Regardless of the prevalence of HIV-1 coinfection, small RNA levels in plasma dropped dramatically before and after therapy. Although miR-29a-3p, SNORD61, miR-17-3p, and miR-133a levels were reduced among persons who reacted to medicine compared to those who did not, no single miRNA or combination of small RNAs was shown to be a significant predictor of successful TB therapy (Wang et al., 2018). To investigate whether there was a similar profile of differential miRNA expression across trials from patients with active TB and healthy controls, samples from patients with active TB were compared to those of healthy controls. Since the past decade or so, researchers have used this method to find miRNA markers in serum/plasma and blood cells. miRNAs described employing broad-spectrum unbiased procedures such as small RNA-seq will likely provide new accurate results than researchers who just concentrate on a few possible miRNAs. Because only a few miRNAs are available in the signature revealed by two or more studies, there is a lack of consistency in the outcomes of such screenings. An array of patient demographics and different types of RNA-seq and microarrays may be at fault. It is thought that these miRNAs play an essential role in TB pathogenesis by decreasing the host’s innate and acquired immune response to intracellular infections, both directly and indirectly. Anti-inflammatory miRNAs, such as miR-21-5p and miR-146a-5p, may also be used to discriminate among active TB and LTBI or an otherwise wholesome condition (Spinelli et al., 2013). As a putative biomarker of active TB, the *M. tuberculosis* inducing miR-155-5p, which is overexpressed in sufferers, plays a vital part in host defense (Etna et al., 2018). Role of different miRNAs in the pathogenesis of tuberculosis is tabulated in **Table 1**.

**Table 1:** Role of different miRNAs in the pathogenesis of tuberculosis.

miRNA	Mode of miRNA expression	Function	Regulated pathway	References
**miR-27b**	Downregulation	Inflammatory responses and apoptosis	TLR-2/MyD88/NF-κB signaling pathway, p53-reactive oxygen species (ROS) signaling pathway.	(Marcinowski et al., 2012; Liu Y et al., 2016) (Liang et al., 2018)
**miR-125a-3p (miR-125a)**	Upregulation	Inhibition of autophagy activation and phagosomal maturation of *Mycobacterium tuberculosis* in the host innate immune cells.	Inhibition of IFN-induced JAK-STAT signaling	(Niu et al., 2018) (Kim et al., 2015) (Yang et al., 2022)
**miR-155 and miR-31**	Upregulation	Inhibits IFN-γ-induced autophagy	WNT and sonic hedgehog signaling	(Yang et al., 2015)
**miR-708-5p**	Downregulation	Mycobacterial vitality and the secretion of inflammatory factors.	Unknown	(Li and Zhang, 2019)
**miR-99b**	Upregulation	Overexpression inhibited TNF-α and IL-6 production	p38/miRNA/NF-κB pathway	
**miR-146a and miR-146a-5p**	Downregulation	Ameliorates inflammation represses Mycobacteria-induced inflammatory response and facilitates bacterial replication, protects against LPS-induced inflammatory injury	TRAF6/NF-κB Pathway Targeting IRAK-1 and TRAF-6	(Jieying et al., 2017) (Chen et al., 2017, Zhang C et al., 2017) (He et al., 2018) (Li et al., 2016) (Zhang et al., 2019) (Li et al., 2013) (Chen et al., 2019) (Wang et al., 2019) (Sharma et al., 2015)
**miR-194**	Upregulation	Lipopolysaccharide- induced inflammatory response	Targeting TNF receptor-associated factor 6 (*TRAF6*)	(Kong et al., 2018)
**miR-18b-5p**	Downregulation	Downregulation favors *Mycobacterium tuberculosis* clearance in macrophages *via* HIF-1α by promoting an inflammatory response	phosphorylation of p38 MAPK and NF-κB p65 was activated by the miR-18b inhibitor.	(Zhu et al., 2021)
**miR-21-5p**	Upregulation	Regulates mycobacterial survival and inflammatory responses	Targeting Bcl-2 and TLR4 signaling	(Nara et al., 2019)
**miR-223-3p**	Upregulation	Promotes the production of pro-inflammatory cytokines, interleukin (IL) 6, IL-1β, and tumor necrosis factor (TNF)-α	Its downregulation resulted in the activation of STAT3	(Lu et al., 2010; Wu M et al., 2019; Wu J et al., 2019)
**miR-2909**	Upregulation	Promotes the production of pro-inflammatory cytokines, interleukin (IL-6, IL-1β, and tumor necrosis factor (TNF)-α	Toll-like receptor (TLR) 4/TLR2/nuclear factor (NF)-κB/signal transducer and activator of transcription (STAT) 3 signaling pathway	(Wu et al., 2019)
**miR-125a**	Upregulation	Enhances erythroid differentiation arrest	Modulates NF-κB Activation	(Kim et al., 2015; Wang et al., 2020) (Yang et al., 2022)
**miR-26b**	Upregulation	Inhibits the immune response to *Mycobacterium tuberculosis* infection	targeting TGFβ-activated kinase-1 (TAK1), a promoter of the NF-κB pathway	(Zhao et al., 2014; Li et al., 2018)
**miR‐140**	Downregulation	Modulates the inflammatory responses	targeting TRAF6	(Li et al., 2019) (Huang et al., 2019)
**miRNA-206**	Upregulation	Regulates the secretion of inflammatory cytokines	TIMP3	(Fu et al., 2016)
**miRNA-124**	Upregulation	Function as an inflammatory regulator and drives the expression of MMP9	Negatively regulates TLR signaling	(Lang and Ling 2012; Ma et al., 2014)
**miRNA-32-5p**	Upregulation	Regulates mycobacterial survival and inflammatory responses	TLR-4/miRNA-32-5p/FSTL1 signaling	(Zhang et al., 2017)
**miRNA-23a-5p**	Upregulation	Modulates mycobacterial survival and autophagy during *Mycobacterium tuberculosis* infection	TLR2/MyD88/NF-κB pathway by targeting TLR2	(Chen et al., 2020)
**miRNA-1178**	Upregulation	Regulates mycobacterial survival and inflammatory responses in *Mycobacterium tuberculosis*-infected macrophages	Negatively regulated the expression of TLR4	(Shi et al., 2018)
**miR-21**	Upregulation	Reported to induce anti‐inflammatory responses	Downregulated the Toll‐like receptor (TLR)/NF‐κB signaling *via* reducing the expression of TNF receptor‐associated factor 6 (TRAF6)	(Zhao et al., 2019)
**miR-325-3p**	Upregulation	Facilitates immune escape of *Mycobacterium tuberculosis*	Targeting LNX1 *via* NEK6 accumulation to promote antiapoptotic STAT3 signaling	(Fu et al., 2020)
**miR-27a**	Upregulation	Alleviates LPS-induced acute lung injury in mice *via* inhibiting inflammation and apoptosis	Modulating TLR4/MyD88/NF-κB pathway	(Zhu et al., 2018)
**miR-148a**	Upregulation	Upregulation of miR-148a inhibits mycobacterial intracellular survival	TLR4/NF-κB signaling pathway, suppresses tuberculous fibrosis by targeting NOX4 and POLDIP2.	(Woo et al., 2022)
**miR-194**	Upregulation	regulates palmitic acid-induced toll-like receptor 4 inflammatory responses	Activate TLR4 signal pathway	(Kong et al., 2018)
**miR-214**	Upregulation	Promotes the calcification through the acceleration of inflammatory reactions	Activate MyD88/NF-κB signaling	(Zheng et al., 2019; Zhao L et al., 2019)
**miR-3473**	Upregulation	Effect inflammatory reactions	Enhances NF-κB *via* targeting TRAF3	(Fang et al., 2016)
**miR-33**	Upregulation	Reprograms autophagy	NF-κB	(Ouimet et al., 2016)
**miR-10b**	Upregulation	Promotes apoptosis	*Via* JNK pathway	(Qin et al., 2018)
**miR-125b**	Upregulation	Blocks TNF synthesis	*Via* MAPK-activated protein kinase 2	(Rajaram et al., 2011)
**miR-138**	Upregulation	Induces apoptosis	Regulates MLK3/NK/MAPK pathway	(Ren et al., 2018)
**miR-517**	Upregulation	Induces oxidative stress	Inactivates JNK pathway	(Yang et al., 2020)
**miR-4268**	Upregulation	Inhibits cell proliferation	*Via* AKT/JNK signaling by targeting Rab6B	Zhao L et al., 2020
**miR-378d**	Upregulation	Clearance of mycobacterial infection	Increases Rab10 expression	Zhu et al., 2020

miRNAs role in the various pathogenesis pathways during tuberculosis infection: Tumor necrosis factor (TLR); Myeloid differentiation primary response protein-88 (MyD88); Nuclear factor-kappa-beta (NF-kB); Interferon (IFN); Janus kinase/signal transducers and activators of transcription (JAK-STAT); Wingless-related integration site (WNT); Interleukin-1 receptor-associated kinase (IRAK); Tumor necrosis factor receptor–associated factor (TRAF); Mitogen-activated protein kinase (MAPK); B-Cell Leukemia/Lymphoma 2 (Bcl2); Transforming growth factor-β-activated kinase 1 (TAK-1); Follistatin-like 1 (FSTL1); Serine/threonine-protein kinase Nek6; E3 ubiquitin-protein ligase LNX; Nicotinamide adenine dinucleotide phosphate oxidase (NOX); Mixed lineage kinase domain (MLK); protein kinase B (AKT); Member rat sarcoma family (Rab).

## Adenosine monophosphate-activated protein kinase/mammalian target of rapamycin signaling pathway

**Figure 2:**
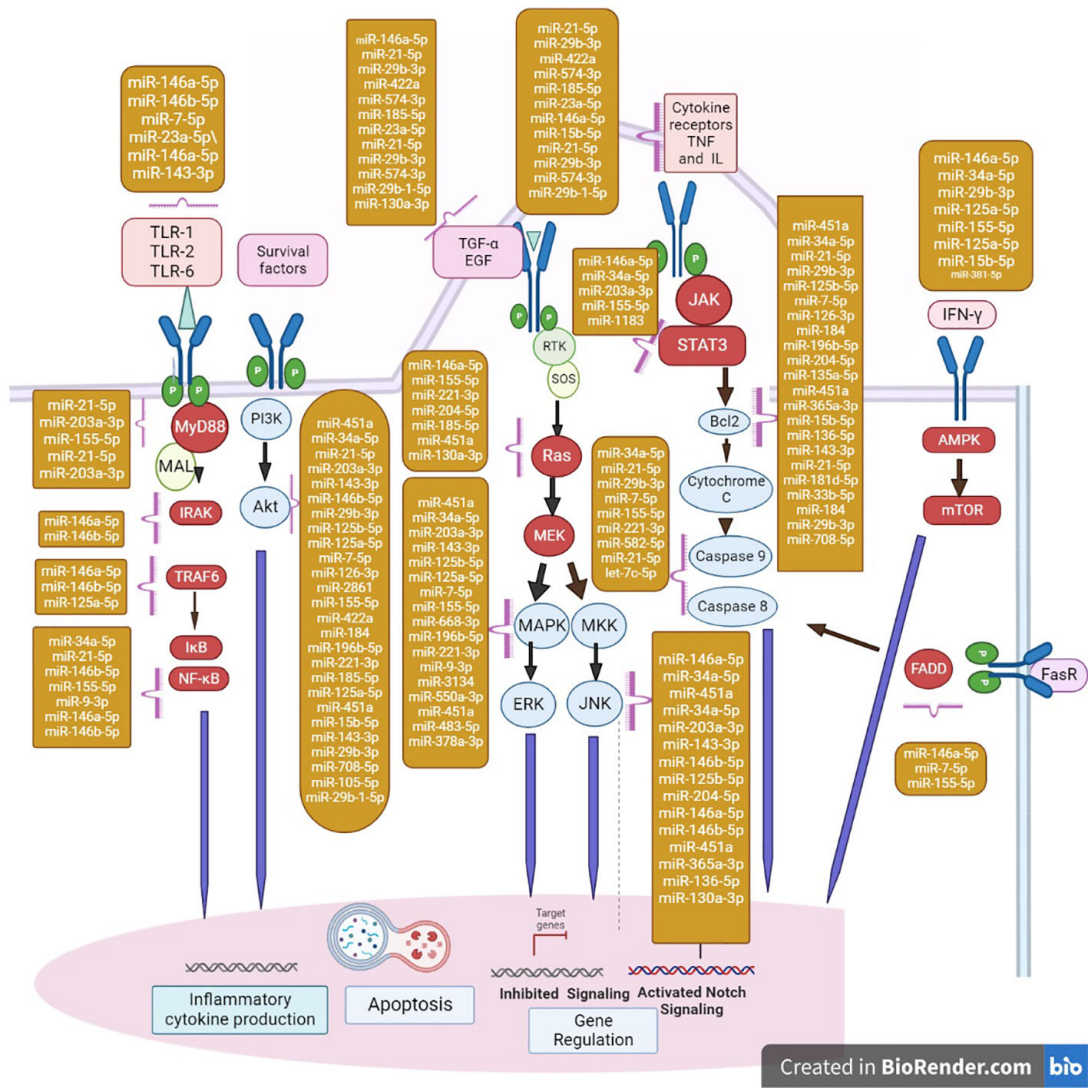
Major miRNAs that regulate apoptosis effectors are shown in the diagram in the yellow box. FasR, Fas Receptor; FADD, Fas-associated death domain protein. miRNAs regulate the major cascades of autophagy. The action of miRNAs involved in the regulation of key members of autophagy cascades; repression/activation of mRNA are shown in the nucleus. mTORC1 induces and regulates the autophagy by miRNAs. AMPK-mTORC1 lies at the heart of regulation of autophagy by integrating numerous stimuli and pathways into a signal for the starting point of autophagy. In addition, ER stress and ROS regulate autophagy independently of the AMPK-mTORC1 pathway. In TNF-α-induced necroptosis, the engagement of TNFR1 recruits Complex I (composed of TRADD, TRAF2). This complex promotes the NF-κB activation and promotes cell survival and inhibits apoptosis. When growth factor receptors are activated, the class I PI3K complex and a small GTPase, Ras, are activated, which activate the PI3K-PKD1-AKT and Ras-Raf-1-MEK1/2-ERK1/2 pathways, respectively. Both AKT and ERK1/2 phosphorylate and inhibit tuberous sclerosis complex, thereby stabilizing Ras homolog, which activates mTORC1, resulting in autophagy inhibition. JNK1-mediated Bcl-2 phosphorylation reduces the binding activity of Bcl-2 and Bcl-xL to initiate autophagy, which promotes cell survival. TLR signaling and miRNAs form a complex network. TLR recruits adaptor proteins and activates downstream signaling cascades that activate the NF-κB signaling pathway and the MAPK signaling pathway in response to specific microbial recognition. This activation causes inflammatory mediators and miRNA genes to be expressed.

AMP-activated protein kinase (AMPK) is a crucial metabolic sensor that responds to alternations in the cellular AMP/ATP ratio following the activation of catabolic energy production. The AMPK pathway is also activated in response to various bacterial infections and inflammation. Various bacterial antigens activate AMPK signaling cascades associated with host response modulation that can either increase or decrease pathogen survival (Grahame Hardie, 2016; Prantner et al., 2017). Previous research has revealed a wide range of AMPK pathway functions, including the regulation of host signaling and participation in significant events. mTOR kinase phosphorylation was more activated in macrophages than AMPK in a time-dependent manner following *M. tuberculosis* infection (Yang et al., 2014). There is evidence that cytosolic *M. tuberculosis* colocalizes with p62 and LC3, which are autophagic machinery components. Watson et al. 2012 found it in only 30% of total *M. tuberculosis* phagosomes, providing evidence that the majority of intracellular *M. tuberculosis* could inhibit xenophagy activation because of TFEB nuclear translocation downregulation. Activation of mTOR during. *M. tuberculosis* infection raises the levels of miRNA-33 and miRNA-33a (Oneyama et al., 2011). miR-124 reduces cell proliferation through G1 phase cell cycle arrest (Gong et al., 2016; He et al., 2020). Furthermore, overexpression of miR-124 overexpression reduces both cell growth and glucose consumption in cells (Zhao et al., 2017), which is consistent with AMPK downregulation. Increased levels of miR-101a and miR-199a decrease AMPK signaling (Liu et al., 2016b) (Li et al., 2020), and miR-101a can both directly and indirectly inactivate AMPK (Li et al., 2018; Liu et al., 2018). miRNAs that were downregulated during hypoxia are also involved in AMPK signaling, which plays an important role in reducing oxidative stress, autophagy, and apoptosis during hypoxia (Li et al., 2016; Tran et al., 2017; da Cruz et al., 2018; Sun et al., 2018; Zhu et al., 2018; Zhao T. et al., 2020). Starvation, genotoxic stress, hypoxia, ER stress, and reactive oxygen species (ROS) all activate signaling pathways that either initiate or regulate autophagy cascades. AMPK-mTORC1 regulates autophagy by integrating multiple stimuli and pathways into a signal for the ULK complex, which is the starting point for autophagy. Several miRNAs have been identified as regulators of AMPK-mTORC1. miRNAs acts as both positive and negative regulators of the gene expression. Upregulated miRNAs mainly target the signaling pathways associated with pathogenesis during the infection. The miRNAs targeting the various pathways are shown in the **Figure 2**.

## Nuclear Factor-κ β/Tumor necrosis factor receptor associated factor 6 signaling pathway

The host component against pathogenic organisms is NF-κB, which is a regulator of cell pro-inflammatory responses (Zhang Q et al., 2017a). NF-κB has been linked to the emergence of chronic inflammation and bacterial infections. Recent studies have reported that several miRNAs have been associated with the inflammatory responses by regulating the *M. tuberculosis* replication and induced pathogenesis by targeting the TRAF-6 signaling pathways (Cui et al., 2018). The detailed mechanisms have to be illustrated further. TRAF-6 belongs to the TNF receptor protein family, acts as an important regulator in many cellular pathways and regulates signal transduction of the TNF receptor superfamily (Chen et al., 2020). TRAF-6 also acts as a link among IRAK-1/IRAK and NF-κB/IB kinase signaling pathways in response to pro-inflammatory cytokines by binding with TGF-β-activated kinase-1 (TAK1) and supporting IκB kinase phosphorylation, ubiquitination, and deterioration after the stimulation of many innate immunity-associated genes (Li H. et al., 2018). During the *M. tuberculosis* infection, upregulation of miRNA-125a in macrophages depends on TLR4 signaling by targeting TRAF-6 and modulating NF-κB (Cui J et al., 2022a; 2022b). This mechanism leads to the attenuation of the immune response and enhances the survival of mycobacteria. The NF-κB pathway is associated with bacteria–host interactions (Westermann, 2018). By identifying the PI3K-AKT-mTOR signaling pathway (PTEN), miR-26b tends to promote the LPS-induced NF-κB signaling pathway and enhances the expression of pro-inflammatory factors (Huang et al., 2012). Studies showed that genetic disruption of the p50 subunit of NF-κB restricts the *M. tuberculosis* infection. Pharmacologic regulation of NF-κB activation decreases the viability of intracellular mycobacteria (Liu et al., 2016a). NF-κB inhibition increases the apoptosis of macrophages and autophagy, which is the established defense mechanism (Bai et al., 2013). Inactivation of NF-κB downregulates the expression of PTEN that regulates cellular activities that may be crucial for pathogen resistance. PTEN signaling regulates infection by affecting various intracellular mycobacterial pathogens (Fang et al., 2016). PTEN deficiency renders susceptibility to infection in multiple cells infected with mycoplasma and *Mycobacterium*. PTEN’s lipid phosphatase activity is critical for infection tolerance. *Mycobacterium* infectious disease activates Akt phosphorylation, and suppression of Akt or PI3K activity regulates cellular infection (Huang et al., 2012). *M. tuberculosis*-infected macrophages secrete cytokines, showing an effective defense mechanism against the pathogen. NF-κB and mitogen-activated protein kinase (MAPK) signaling pathways regulate the expression of various cytokines (Gañán-Gómez et al., 2014). Cytokines such as TNF-α, IL-6, and IL-1β are potent mediators showing immune response against the *M. tuberculosis* bacilli (Cui et al., 2021). Targeting the cytokines and their regulatory pathways restricts the host immune response. For example, *M. tuberculosis* virulence protein PtpA arrests the NF-κB and JNK signaling pathways (Rothchild et al., 2016). Treatment of cells with early secreted antigenic target-6 (ESAT-6) prevents TLR-associated NF-κB activation (Yang et al., 2015). TAK is a serine/threonine protein kinase associated with the activation of NF-κB pathway. Studies show that miRNAs regulate TAK expression to promote chemoresistance. Upregulation of miR-143 attenuates the function of TAK. miR-146a and miR-26b also target the TAK to promote apoptosis and are associated with the NF-κB pathway inhibition. miR-143 and miR-146a inhibit the NF-κB signaling pathway (Chen Y et al., 2016; Chen et al., 2019).

## Toll-like receptor signaling

During the *M. tuberculosis* infection, TLR2-deficient animals were more susceptible than control mice, but TLR2- and TLR4-deficient mice were as vulnerable as control mice in a low-dose *M. tuberculosis* challenge (Ju et al., 2018). Pattern recognition receptors (PRRs) expressed on leukocytes activate phagocytosis and host defense mechanisms through the promotion of signaling cascades. TLRs and mannose receptors associated with PRRs play a critical role in immune response and detect the pathogen-derived molecules. Most of the mycobacterial antigens act as agonists for TLRs (Liu Y. et al., 2016). Inoculation of BCG was believed to be dependent on TLR2 and TLR4. Most of the mycobacterial proteins and lipids are associated with the TLR-dependent signaling cascades. TLR regulates hundreds of the host genes that are associated with signaling and acts against microbial antigens, so studying about TLR molecular mechanisms has great importance (Shariq et al., 2021). TLR signaling was negatively affected by higher levels of miRNA-146a-5p, miR-21-5p, miR-99b-5p, and miR132-5p 9 (Wu et al., 2012). The overexpression of miR-27a-5p and miR-33 in *M. tuberculosis*-infected cells inhibits the creation of autophagosomes and the killing of *M. tuberculosis* by macrophages (Marcinowski et al., 2012). As an additional line of defense against intracellular infections, host miRNAs such as miR-325-3p and miR-20b-5p impact cell death and inflammasome activation (Kumar et al., 2015). miRNAs associated with the host innate and adaptive immune systems, such as miR-155-5p and let-7f, have a role in the activation of signaling pathways during *M. tuberculosis* infection, which is necessary for the clearance of pathogens (Iwai et al., 2015; Li et al., 2016). Although miR-29a-3p, SNORD61, miR-17-3p, and miR-133a levels were reduced among people who reacted to medicine compared to those who did not, no single miRNA or combination of small RNAs was shown to be a significant predictor of successful TB therapy (Dersch et al., 2017). *M. tuberculosis*-mediated TLR2/1 signaling increases the expression of the vitamin D receptor and the vitamin D hydroxylase, resulting in enhanced production of antimicrobial peptides (Lv et al., 2017). TLR4 may contribute to *M. tuberculosis* resistance; however, no agreement has been achieved at this point. TLR4 has a protective role in adaptive immunity against pulmonary TB *in vivo*; the non-functional TLR4 causes high mortality and increased bacterial burden in the lungs. miR-146a-5p, miR-21-5p, miR-99b-5p, and miR-132-5p are highly expressed in TB patients and adversely regulate host signaling cytokines in myeloid cells triggered by TLR signaling, promoting *M. tuberculosis* survival (He et al., 2018). Other miRNAs that are upregulated in *M. tuberculosis*-infected macrophages, such as miR-27a-5p, miR-33, miR-125-5p, and miR-144-5p, inhibit autophagy formation and *M. tuberculosis* killing by macrophages. Both miR-29a-3p and miR-125-5p are upregulated in infected macrophages and directly target IFN and TNF, thereby reducing the immune reaction to intracellular *M. tuberculosis* (Stepanov et al., 2015). Cell necrosis and inflammasome formation are two other mechanisms of defensive strategy against intracellular pathogens that are controlled by *M. tuberculosis*-induced host miRNAs such as miR-325-3p and miR-20b-5p. However, some miRNAs that are influenced during *M. tuberculosis* infection, such as miR-155-5p and let-7f, play a crucial role in the activation of host innate and adaptive immunity, as well as microbial clearance (Sinigaglia et al., 2020).

## Interleukin-1 receptor-associated kinase-1 family pathway

Ligand identification activates the TIR-containing adaptor receptor MyD88, which further binds to IRAK-1 and IRAK-4 (Li et al., 2013; Gu et al., 2017). IRAK has a destruction domain and a serine/threonine kinase domain, and there are four members of the IRAK family: IRAK-1, IRAK-2, IRAK-M, and IRAK-4 (Li et al., 2002). Studies have revealed that IRAK-4 functions upstream of IRAK-1 in the TLR complex (Zhang et al., 2019). Mutations in the *IRAK-4* gene have been linked to a higher sensitivity to bacterial infection in patients with Mendelian susceptibility to mycobacterial disease, and *M. tuberculosis*-infected patients are resistant to TLR ligands (Cui et al., 2018). Moreover, NF-κB essential modulator (NEMO) and IRAK-4 were revealed to be important in IL-12 formation and increased IFN-γ production in humans and mice, which would be vital to creating protective immune responses against mycobacterial infection (Wu et al., 2019). In response to TLR stimulation, IRAK-4 associates with IRAK-1, and the emergence of a dominant negative form of IRAK-4 negative regulator IRAK-1 activation (Li S et al., 2002). IRAK-deficient mice secrete more cytokines in response to TLR ligands (Lomaga et al., 1999). IRAK-M is the negative regulator of the TLR signaling that shows the important role of this protein in suppressing mycobacteria-induced inflammasome activation and TLR signaling pathways. The MAPK pathways are triggered by primary stimulations such as mycobacterial products or whole mycobacteria, resulting in the stimulation of transcription factors such as NF-κB and activator protein-1 (AP-1) (Wang et al., 2001). miRNAs regulating the IRAK signaling pathway were shown in **Table 2**.

**Table 2:** miRNAs associated with regulating the various signaling pathways (Source miRbase software).

miRNA		P-Value	Targets
**hsa-miR-146a-5p**	Experimental (any)	5.53E-06	FADD,IL6,IRAK1,IRAK2,ITGB2,NFKB1, RHOA,STAT1,TGFB1,TLR2,TLR4,TRAF6
**hsa-miR-451a**	Experimental (any)	7.44E-05	AKT1,BCL2,IL6,MAPK1,RAB5A
**hsa-miR-34a-5p**	Experimental (any)	1.60E-04	AKT1,APAF1,BAX,BCL10,BCL2,CASP10, CASP3,CASP8,CASP9,CYCS,IFNB1,IL10, MAPK3,NFKB1,PPP3R1,RIPK2,SRC, STAT1,TNF,TNFRSF1A
**hsa-miR-21-5p**	Experimental (any)	7.89E-04	AKT2,APAF1,ARHGEF12,BCL10,BCL2,CASP8, CEBPB,IL12A,IL1B,IRAK1,LAMP2,MALT1, MYD88,NFKB1,TGFB1,TGFB2,TLR4
**hsa-miR-203a-3p**	Experimental (any)	0.001	AKT2,CREB1,IL6,MAPK8,MAPK9,MYD88, NFYA,SRC,STAT1,SYK,TNF
**hsa-miR-143-3p**	Experimental (any)	0.001	AKT1,AKT2,BCL2,CALM3,IL10RB, MAPK1,NFYB,TLR2,TNF
**hsa-miR-146b-5p**	Experimental (any)	0.002	AKT3,IL6,IRAK1,NFKB1,RHOA,TLR4,TRAF6
**hsa-miR-29b-3p**	Experimental (any)	0.003	AKT2,AKT3,BCL2,CALM3,CASP8, IFNG,TGFB1,TGFB2,TGFB3
**hsa-miR-125b-5p**	Experimental (any)	0.015	AKT1,BCL2,CEBPG,CREBBP,HSPD1,JAK2, MAPK14,RAF1,SPHK1,TNF,VDR
**hsa-miR-125a-5p**	Experimental (any)	0.015	AKT1,BCL2,IFNG,JAK2,MAPK14, MAPK8,RAF1,TRAF6
**hsa-miR-7-5p**	Experimental (any)	0.016	AKT3,ARHGEF12,BAX,BCL2, CALM3,CAMK2D,CASP9,FADD, MAPK9,NFYA,RAF1,RELA,TLR4
**hsa-miR-126-3p**	Experimental (any)	0.016	AKT1,AKT2,BCL2
**hsa-miR-2861**	Experimental (any)	0.017	AKT2,APAF1,CYCS,VDR
**hsa-miR-155-5p**	Experimental (any)	0.024	AKT1,APAF1,ATP6V1H,CASP3,CEBPB, FADD,HLADPA1,IFNGR1,IL6,MAPK13, MAPK14,MYD88,NFKB1,NFYC, RAB5C,RHOA,STAT1
**hsa-miR-422a**	Experimental (any)	0.024	AKT1,TGFB1,TGFB2
**hsa-miR-184**	Experimental (any)	0.025	AKT1,AKT2,BCL2
**hsa-miR-668-3p**	Experimental (any)	0.027	MALT1,MAPK1,MAPK14
**hsa-miR-601**	Experimental (any)	0.03	ATP6AP1,CREBBP
**hsa-miR-196b-5p**	Experimental (any)	0.033	AKT1,BCL2,CALM1,CALM3,MAPK1
**hsa-miR-221-3p**	Experimental (any)	0.037	AKT3,APAF1,CASP3,CORO1A,MAPK10, NFYA,NFYC,RAB5C,RHOA
**hsa-miR-9-3p**	Experimental (any)	0.038	MAPK1,MAPK3,NFKB1,PPP3R1
**hsa-miR-574-3p**	Experimental (any)	0.039	EP300,TGFB1
**hsa-miR-204-5p**	Experimental (any)	0.041	BCL2,BID,CAMK2G,CREB1, HLA-DRB1,HLA-DRB5,IL1B,JAK2,PPP3R1,RAB5B
**hsa-miR-135a-5p**	Experimental (any)	0.042	BCL2,JAK2,SRC,TRAF6
**hsa-miR-185-5p**	Experimental (any)	0.048	AKT1,ATP6AP1,CALM3,CAMK2D,CEBPB, IL10RA,RAB5B,RHOA,TGFB1
**hsa-miR-3134**	Experimental (any)	0.048	MAPK13,RAF1,RELA
**hsa-miR-23a-5p**	Experimental (strong)	3.72E-05	APAF1,LAMP1,TGFB2,TLR2
**hsa-miR-146a-5p**	Experimental (strong)	5.12E-05	FADD,IL6,IRAK1,IRAK2,NFKB1,RHOA, STAT1,TGFB1,TLR2,TLR4,TRAF6
**hsa-miR-146b-5p**	Experimental (strong)	6.39E-04	IL6,IRAK1,NFKB1,TLR4,TRAF6
**hsa-miR-550a-3p**	Experimental (strong)	0.001	MAPK1,MAPK3
**hsa-miR-125a-5p**	Experimental (strong)	0.002	AKT1,BCL2,IFNG,JAK2, MAPK14,RAF1,TRAF6
**hsa-miR-451a**	Experimental (strong)	0.003	AKT1,BCL2,IL6,MAPK1,RAB5A
**hsa-miR-582-5p**	Experimental (strong)	0.004	CASP3,CASP9,CREB1
**hsa-miR-365a-3p**	Experimental (strong)	0.006	BAX,BCL2,IL6
**hsa-miR-15b-5p**	Experimental (strong)	0.009	AKT3,BAX,BCL2,IFNG,TGFB1
**hsa-miR-136-5p**	Experimental (strong)	0.012	BCL2,IL6
**hsa-miR-143-3p**	Experimental (strong)	0.017	AKT1,AKT2,BCL2,TLR2,TNF
**hsa-miR-21-5p**	Experimental (strong)	0.019	AKT2,APAF1,BCL10,BCL2,CASP8,CEBPB, IL12A,IL1B,IRAK1,MYD88,TGFB2
**hsa-miR-181d-5p**	Experimental (strong)	0.02	BCL2,MALT1
**hsa-miR-33b-5p**	Experimental (strong)	0.023	BCL2,CREB1,SRC
**hsa-miR-203a-3p**	Experimental (strong)	0.024	AKT2,CREB1,MYD88,SRC,STAT1,TNF
**hsa-miR-184**	Experimental (strong)	0.025	AKT1,AKT2,BCL2
**hsa-let-7c-5p**	Experimental (strong)	0.027	CASP3,CEBPB,IL10,IL6
**hsa-miR-29b-3p**	Experimental (strong)	0.027	AKT2,AKT3,BCL2,IFNG, TGFB1,TGFB2,TGFB3
**hsa-miR-574-3p**	Experimental (strong)	0.028	EP300,TGFB1
**hsa-miR-483-5p**	Experimental (strong)	0.029	MAPK3,RHOA
**hsa-miR-708-5p**	Experimental (strong)	0.031	AKT1,AKT2,BCL2
**hsa-miR-105-5p**	Experimental (strong)	0.033	AKT1,TLR2
**hsa-miR-9-3p**	Experimental (strong)	0.033	MAPK1,MAPK3,NFKB1
**hsa-miR-29b-1-5p**	Experimental (strong)	0.034	AKT3,TGFB1
**hsa-miR-130a-3p**	Experimental (strong)	0.041	IL18,RAB5A,TGFB1,TNF
**hsa-miR-378a-3p**	Experimental (strong)	0.043	KSR1,MAPK1,TGFB2
**hsa-miR-146a-5p**	Experimental (any)	7.13e-4	IFIT1,IFIT3,IFITM1,STAT1
**hsa-miR-381-5p**	Experimental (any)	0.006	IFNAR1,IFNAR2
**hsa-miR-503-3p**	Experimental (any)	0.008	IFITM1,SOCS1
**hsa-miR-373-3p**	Experimental (strong)	0.019	IRF9,JAK1
**hsa-miR-1183**	Experimental (any)	0.026	PIAS1,STAT1

## Fas-associated death domain-containing protein signaling

Death ligands such as Fas ligand (FasL) bind to death receptors in the FasR receptor. Following this interaction, the death-inducing signaling complex (DISC) is formed, which includes the Fas-associated death domain-containing protein (FADD) and procaspase-8/10. RNAi-mediated FADD knockdown in cells reduced NF-κB signaling. Exogenous FADD expression prevented NF-κB signaling (Dockrell, 2003). FADD loss-of-function mutations in the death effector inhibited the caspase-8 and NF-κB activation that promotes apoptosis. Caspase-8 deficiency inhibited TNF-related apoptosis-inducing ligand (TRAIL)-induced NF-κB activation. These findings reveal a mechanism for TRAIL-induced NF-κB activation that involves the TRAIL receptors DD, FADD, and caspase-8. These proteins play an important role in apoptosis signaling and are the mediators of non-apoptotic CD95 signaling during T-cell proliferation (Welz et al., 2011). FADD-deficient T cells show reduced proliferation, implying that FADD plays an important role in proliferation signaling. FADD can be regulated transcriptionally by miR-155 (Wang et al., 2011) or miR-128a. miR-128a ectopic expression conferred Fas resistance in cells by directly targeting FADD, but antagonizing miR-128a function made cells susceptible to Fas-mediated apoptosis (Yamada et al., 2014).

## Janus kinase/signal transducers and activators of transcription signaling

Following the binding of activated calcium ions to cAMP, they activate small Ras-like GTPases like Ras-proximate-1 (Rap1), which is primarily involved in cell adhesion and junction formation during cell proliferation. cAMP is also known to increase ERK1/2 phosphorylation *via* ROS-dependent activation of Ras. Through the negative feedback regulation, miR-146 plays an important role in the control of TLRs and cytokine signaling (Brooks et al., 2014). miRNAs have the ability to regulate the levels of molecules by being involved in the negative feedback of PRR-induced signaling (Mao et al., 2005). miR-124 was discovered to be a negative regulator of inflammation by targeting several pathways, including signal transducer and activator of transcription (STAT) and TLRs (O’Shea et al., 2013). miR-124 inhibits intestinal inflammation by attenuating the production of IL-6 and TNF-α *via* targeting STAT3, a major factor in inflammatory response, and acetylcholinesterase, a negative regulator of the cholinergic anti-inflammatory signal. Sun et al. (2018) reported that miR-124 inhibits STAT3 to reduce IL-6 production and TNF-α-converting enzyme to inhibit TNF-α release in response to LPS. Lower levels of miR-124 and higher levels of STAT3 promote inflammation and disease pathogenesis. miR-124 expression is increased in pulmonary TB patients. miR-124 negatively regulates multiple TLR signaling components, including TLR6, MyD88, TNF-α, and TRAF6, implying an underlying negative feedback loop between miR-124 and TLR signaling to prevent excessive inflammation (Wang S. et al., 2018). In both calves and humans, a decrease in miR-124 expression contributes to high proliferation and pulmonary inflammation.

## Myeloid differentiation primary response protein (MyD88) signaling

MyD88 is an intracellular molecule connected to IRAK and TLRs to transduce signals. MyD88 activates MAPK, PI3K, NF-κB, and IRAK following the initiation of the signal cascade. MyD88 deficiency impairs the macrophage response to bacterial antigens and makes the individual susceptible to infection. However, macrophages activate antibacterial immunity through MyD88-independent mechanisms (Cervantes, 2017). MyD88 deficiency improved resistance to polymicrobial sepsis, indicating that both MyD88-dependent and MyD88-independent antibacterial mechanisms exist. Many studies that showed the regulation of individual genes in macrophages by subcellular microbial products through the TLR/MyD88 signal transduction pathway have been conducted (Huang et al., 2019). However, there appears to be no study showing the role of MyD88 in macrophage activation showing antimicrobial activity. Three unexpected findings emerged in MyD88-deficient mice, implying that the current understanding of macrophage activation needs to be revised. Macrophages undergo active self-priming activation, which is dependent on MyD88. MyD88 is not involved in the IFN-γ signaling pathway; however, the expression of many genes in macrophages in response to IFN-γ is mostly dependent on MyD88. The majority of transcriptional responses of macrophages against *M. tuberculosis* do not require MyD88. This suggests that TLRs are not the primary receptors for recognizing *M. tuberculosis* or that TLR-dependent responses are mediated by MyD88-independent signaling pathways (O’Connell et al., 2010; Sharbati et al., 2011). miR-155 modulates the production of inflammatory mediators in response to microbial stimuli by negatively regulating the expression of an important TAK1- and TRAF6-binding protein 2 (TAB2) (Ceppi et al., 2009). Additionally, miR-146a inhibits TLR signaling, thereby inhibiting the production of inflammatory mediators (Taganov et al., 2006; Chen et al., 2007). When PAMPs are recognized, TLR signaling is activated, which leads to the transcriptional activation of genes encoding pro-inflammatory mediators following the activation of antigen-specific adaptive immune response *via* a MyD88-dependent or -independent pathway (Medzhitov et al., 1998). Various signalling pathways are regulated by more than one miRNAs. The miRNAs that regulate the various signalling pathway during the tuberculosis infection are shown in the **Table 2**.

## B-Cell Leukemia/Lymphoma 2 pathway

The Bcl-2 family members that promote and prevent apoptosis are controlled differently by *M. tuberculosis*. The prototypical antiapoptotic protein Bcl-2 has homologs in the Bcl-2 family (Klingler et al., 1997). The antiapoptotic family member bfl-1 is upregulated in macrophages during the *Mycobacterium bovis* BCG infection (Perskvist et al., 2002). An antiapoptotic gene called Bcl-xL was upregulated during *M. tuberculosis* infection after Bcl-2 was downregulated (Mogga et al., 2002). Infection with *M. tuberculosis* causes neutrophils to produce more of the proapoptotic family protein Bcl-2-associated X-protein (Bax) and less of the antiapoptotic family protein Bcl-xL (Harris and Thompson, 2000). Bcl-2 was upregulated and Bax was downregulated in animal models of TB. By reducing neutrophil levels and increasing B-cell levels, a number of miRNAs have been linked to the regulation of the apoptotic pathway (van Rensburg et al., 2018). miR-365, which is highly expressed in cells, directly targets the proapoptotic protein Bax, and these interactions are linked to drug resistance in pancreatic cancer cells. By inhibiting Bax expression, miR-125b conferred drug resistance in breast cancer cells (Zhou et al., 2010). By specifically targeting Bcl-xL and inducing apoptosis, miR-491 reduces the viability of cells. Treatment with miR-491 prevents tumor growth in naive mice *in vivo* (Nakano et al., 2010). Downregulation of miR-133a has been linked to tumor development and prognosis. Restoration of miR-133a inhibits cell division and triggers apoptosis. Bcl-xL regulation by miR-608 has also been demonstrated (Zhang et al., 2014). The expression of Bcl-2 was discovered to be inversely correlated with miR-15a and miR-16-1 (Cimmino et al., 2005). These two miRNAs directly inhibit Bcl-2 at the posttranscriptional level, according to a subsequent study, and also cause apoptosis (Zhang Y. et al., 2014). Bcl-2 protein signaling was elevated when miR-204 was downregulated. MiR-148a and miR-24-2c also directly inhibit Bcl-2 expression (Srivastava et al., 2011; Zhang et al., 2011). Apoptosis is regulated by the endogenous miR-23a/b and miR-27a/b inhibitors of apoptotic peptidase-activating factor (Apaf)-1 expression. It has been demonstrated that miR-133 and miR-24a directly repress caspase-9 to regulate cell fate (Xu et al., 2007; Walker and Harland, 2009; Ji et al., 2013; Chen et al., 2014).

## Caspase pathway

Caspases play an important role in classical apoptosis (Kroemer and Martin., 2005). Caspase activation is not necessarily important in all types of apoptosis. Apoptosis can be triggered by the extrinsic pathway and the intrinsic pathway involving ligation of cell surface death receptors through respective ligands and regulating the Bcl-2 family of pro- and antiapoptotic proteins, respectively. Suppression of caspase activity is cytoprotective when cells are stimulated to undergo apoptosis *via* death receptor ligation (Maquarre et al., 2005). Caspases, on the other hand, are terminal effectors of the mitochondrial pathway, and this type of cell death is mostly caspase independent (Jäättelä, 2001; Hudson et al., 2013). Few studies suggest that apoptotic cell death can occur in the absence of caspases or in the presence of both caspase and non-caspase protease activity (Sacconi et al., 2012). TNF-α activates caspases and can initiate several cell death pathways, including lysosomal permeabilization mediated by cathepsin B release, which activates the mitochondrial apoptosis pathway (Guicciardi et al., 2000). Although these caspase-independent pathways have not been fully characterized, calpains and serine proteases have been implicated as cell death mediators (Zhang L. et al., 2012; Guo et al., 2013). Overexpression of miR-337-3p and miR-17-5p/miR-132-3p/-212-3p, respectively, can regulate executioner caspase-3 and caspase-7. Furthermore, miRNA overexpression, particularly miR-337-3p, reduces TRAIL cytotoxicity.

## c-Jun N-terminal kinase/mitogen-activated protein kinase pathways

MAPK-regulated ERK, JNK, and p38 groups alter gene expression. MAPK signaling pathways are activated during mycobacterial infection and are related to mycobacterial pathogenesis (Song et al., 2003; Pasquinelli et al., 2013; Guo et al., 2019). The p38 MAPK pathway is associated with mycobacteria-induced IL-10, and other cytokines such as TNF-α/IL-4/IFN-γ are produced (Bachstetter and Van Eldik, 2010; de Souza et al., 2014). TNF-α expression in human macrophages is increased by ERK1/2 signaling (Surewicz et al., 2004). MAPK signaling pathways are involved in the regulation of antimycobacterial pathways such as phagosome acidification, apoptosis, and antigen presentation *via* MHC class II expression. Previous research indicates that the p38 MAPK pathway could serve as a means for mycobacteria to be suppressed. Suppression of p38 MAPK activity increases phagosome acidification. Inactivation of the p38 MAPK pathway causes an increase in phagosome acidification and a significant increase in monocytes’ ability to kill mycobacteria (Klug et al., 2011). Synthesis of TNF in human macrophages is inhibited by lipomannan from virulent *M. tuberculosis*, but not by avirulent *Myocobacterium smegmatis*. This variation in response is due to TB and lipomannan induces the stimulation causing TNF mRNA transcripts to destabilize following the reduced expression of TNF protein. *Mycobacterium smegmatis* Lipomannan increases MAPK-activated protein kinase 2 (MK2) phosphorylation, which is important for maintaining TNF mRNA stability by contributing miRNAs. miR-125b binds to the 3’ UTR region of TNF mRNA and destabilizes the transcript, whereas miR-155 increases TNF production by increasing TNF mRNA half-life and reducing the expression of SHIP1, which is the negative regulator of the PI3K/Akt pathway (Rajaram et al., 2011). Signaling *via* ERK1/2 and p38 inhibits a well-known mycobacterial TLR2 agonist. Thus, the p38 MAPK and ERK1/2 pathways regulate macrophage antimicrobial function and antigen presentation by infected macrophages, potentially contributing to host immune evasion (Liu P et al., 2016; Liu P et al., 2016; Hölscher et al., 2020). TNF mRNA is stabilized by activated MK2 (Campbell et al., 2014). Non-phosphorylated TTP Tristetraprolin binds to the Adenine/Guanine rich elements ARE region of target mRNAs and causes rapid degradation *via* a variety of mechanisms (Chen et al., 2013). During the *Mycobacterium* infection, TLR2-dependent MAPK p38 and the PI3K/Akt pathway stimulates an increase in TNF mRNA expression. Mycobacteria cause the activation and expression of MK2, miR125b, and miR-155 to differ. TNF expression in mycobacteria-infected macrophages is significantly influenced by MAPK p38 and Akt activation. miR-125b inhibits TNF production by targeting the 3′ UTR of the TNF transcript. It also increases the stability of B-Ras2, an inhibitor of NF-κB signaling in human macrophages, lowering the inflammatory response (Niu et al., 2018). TNF production is regulated by hsa-miR-155, which targets the inositol phosphatase for degradation *via* its 3′ UTR interaction (Yang et al., 2015). Mycobacteria cause differential expression of miRNAs, which are involved in mRNA signal transduction in human macrophages (Schifano et al., 2017). The JNK signaling pathway is important in many biological processes, including embryogenesis. These kinases regulate the expression of host genes involved with apoptotic cell death pathways and carcinogenesis, thereby controlling the functions of neurons and the immune system. Several miRNAs and long noncoding RNAs (lncRNAs) are functionally related to JNKs (Ghafouri-Fard et al., 2021). miR-138 targets mixed-lineage kinase-3 (MLK3), an important component of the JNK/mitogen-activated kinase pathway. miR-138 upregulation diminished proapoptosis factors and apoptosis rate. Upregulation of miR-138 decreased the expression of JNK, phosphorylated JNK (p-JNK), c-jun, p38 MAPK, p-p38 MAPK, iNOS, and COX-2 (Ghafouri-Fard et al., 2021). Low concentrations of MLK3 proteins and inhibition of the JNK/MAPK signaling pathways provide protection. miRNA-363-3p transcriptional regulation is mediated by DNA methylation. The dual-specificity phosphatase 10 targets miRNA-363-3p, and its inhibition promotes JNK phosphorylation. The miRNA-363-3p/DUSP10/JNK axis was linked to the inhibition of homologous recombination and DNA repair pathways. An innovative therapy is thought to be the miRNA-363-3p/DUSP10/JNK axis (Zhou et al., 2022). miR-517a controls oxidative stress. miR-517a suppression enhances cleaved caspase-3 expression, Bax/Bcl-2 ratio, ROS and MDA levels, and cell apoptosis while decreasing ERK1/2 phosphorylation, T-AOC levels, SOD activity, cell proliferation, and mitochondrial membrane potential. Lower levels of miR-517a result in the inactivation of the JNK signaling pathway. As a result, melanoma cells experience increased oxidative stress (Tofannin et al., 2011). miR-221 demonstrates the impact of cyclin-dependent kinase inhibitor on the occurrence and progression of cell cycle progression (Sun et al., 2011). miRNA-31 identifies the cell division cycle protein 42 and forms a negative feedback chain for JNK inactivation upon the formation of miR-31/Cdc42/phosphorylated MLK3 (p-MLK3). miR-31 and p-JNK were found in high concentrations in the liver tissues of Drug induced lung injury patients with various causes. miR-31 can inhibit the overactivation of the ROS/JNK/mitochondrial diseased death loop in Acetaminophen-induced DILI hepatocytes, suggesting a new therapeutic potential for JNK overactivation-based liver injury. The triggering of apoptosis was mediated. Lower levels of miR-517a result in the inactivation of the JNK signaling pathway. As a result, melanoma cells experience increased oxidative stress (Tofannin et al., 2011). miR-221 demonstrates the impact of cyclin-dependent kinase inhibitor on the occurrence and progression of cell cycle progression (Sun et al., 2011). miRNA-31 identifies the cell division cycle protein 42 and forms a negative feedback chain for JNK inactivation upon the formation of miR-31/Cdc42/p-MLK3. miR-31 and p-JNK were found in high concentrations in the liver tissues of DILI patients with various causes. miR-31 can inhibit the overactivation of the ROS/JNK/mitochondrial diseased death loop in APAP-induced DILI hepatocytes, suggesting a new therapeutic potential for JNK overactivation-based liver injury. The triggering of apoptosis was mediated. Apoptosis was induced by activating the JNK pathway and using a JNK specific inhibitor, which was found to entirely inhibit miR-10b-induced apoptosis.

## Conclusion

In this review, we narrated the upregulation and downregulation of miRNAs that target the components of six signaling pathways activated in TB infection. After a careful review of the bibliography, we observed the upregulation and downregulation of miRNAs, playing an important role in the pathways. Most of the signaling pathways remain active during the TB infection. We mentioned the important roles of pathways and their regulating miRNAs. These miRNAs can be considered as therapeutic targets. Therefore, targeting their expression can modulate the activity of signaling pathways. We reviewed the importance of miRNAs, posttranscriptional regulators that control mRNA stability in signaling pathways. miRNAs can multiply and regulate cellular outcomes in response to various extracellular signals by acting as genetic switches or fine-tuners. Signaling networks, on the other hand, control the stability, biogenesis, and abundance of miRNAs over time by regulating layers of the miRNA biogenesis pathway. The detailed study of the miRNAs regulating the immune-associated pathways is useful for the development of miRNA mimetic/inhibitor molecules. Immune effects induced by miRNA drugs are currently the major challenges of miRNA therapeutics.

## Author contributions

KD conceptualized the idea, analyzed the data and wrote the manuscript. DC helped in improving the manuscript. All authors contributed to the article and approved the submitted version.

## Conflict of interest

The authors declare that the research was conducted in the absence of any commercial or financial relationships that could be construed as a potential conflict of interest.

## Publisher’s note

All claims expressed in this article are solely those of the authors and do not necessarily represent those of their affiliated organizations, or those of the publisher, the editors and the reviewers. Any product that may be evaluated in this article, or claim that may be made by its manufacturer, is not guaranteed or endorsed by the publisher.

## Glossary

**Table:**

**FADD**	Fas-associated death domain
**IL**	interleukin
**IRAK**	interleukin-1 receptor-associated kinase
**ITGB**	integrin subunit beta
**NF-κB**	nuclear factor kappa B
**RHOA**	Ras homolog family member A
**STAT**	signal transducer and activator of transcription
**TGF-β**	transforming growth factor beta
**TLR**	Toll-like receptor
**TRAF**	tumor necrosis factor receptor-associated factor
**AKT**	Akt serine/threonine kinase family
**BCL**	B-cell lymphoma
**MAPK**	mitogen-activated protein kinase
**RAB5A**	Ras-related protein Rab-5A
**APAF**	apoptotic peptidase-activating factor
**Bax**	Bcl-2-associated X-protein
**CASP**	Caspase
**CYCS**	cytochrome C somatic
**IFNB1**	interferon
**MAPK**	mitogen-activated protein kinase
**PPP3R**	protein phosphatase 3 regulatory subunit B, alpha
**RIPK**	receptor interacting serine/threonine kinase
**SRC**	proto-oncogene tyrosine-protein kinase Src
**STAT**	signal transducer and activator of transcription
**TNF**	tumor necrosis factor
**TNFRSF1A**	tumor necrosis factor receptor superfamily member 1A
**APAF**	apoptotic peptidase-activating factor
**ARHGEF12**	Rho guanine nucleotide exchange factor
**CEBPB**	CCAAT enhancer binding protein beta
**MALT**	mucosa-associated lymphoid tissue lymphoma translocation protein
**MYD88**	myeloid differentiation primary response 88
**TGFB1**	transforming growth factor-B
**TLR4**	Toll-like receptor-4
**CREB**	CAMP responsive element binding protein
**NFYA**	nuclear transcription factor Y subunit alpha
**SYK**	spleen-associated tyrosine kinase
**CALM**	calmodulin
**RHOA**	Ras homolog family member A (RhoA)
**CEBPG**	CCAAT enhancer binding protein beta
**CREBBP**	CREB binding protein
**HSPD**	heat shock protein D
**JAK**	Janus kinase
**RAF**	RAF proto-oncogene serine/threonine-protein kinase
**SPHK**	sphingosine kinase
**EP300**	E1A-associated protein p300
**KSR**	kinase suppressor of Ras
**IFIT**	interferon-induced protein with tetratricopeptide repeats
**IFITM**	interferon-induced transmembrane protein
**IFNAR**	interferon alpha and beta receptor subunit
**IFITM**	interferon-induced transmembrane protein
**SOCS**	suppressor of cytokine signaling
**IRF**	interferon-regulatory factor
**PIAS**	protein inhibitor of activated STAT
**ARHGEF12**	Rho guanine nucleotide exchange factor 12
**NFYA**	nuclear transcription factor Y subunit alpha
**RELA**	REL-associated protein
**VDR**	vitamin D receptor
**ATP6V1H**	ATPase H+ transporting V1 subunit H
**CEBPB**	CCAAT enhancer-binding protein beta
**HLADPA1**	major histocompatibility complex, Class II, DP alpha 1
**MyD88**	myeloid differentiation primary response 88
**NFYC**	nuclear transcription factor Y subunit gamma
**CORO1**	coronin
**PPP3R1**	protein phosphatase 3 regulatory subunit B, alpha
**EP300**	E1A-associated protein p300
**BID**	BH3-interacting domain death agonist
**CAMK2G**	calcium/calmodulin dependent protein kinase II gamma
**RHOA**	Ras homolog family member A
**LAMP**	lysosomal associated membrane protein
**KLF4**	Kruppel like factor-4
**NLRP3**	NLR family pyrin domain containing 3
**NLR**	Nucleotide-binding domain and leucine rich repeat containing
**NF1A**	Nuclear factor 1 alpha
**Bag2**	Bcl2 associated athanogene 2
**FOXO3**	Forkhead box O3
**PI3K**	Phosphoinositide-3-Kinase
**mTOR**	Mammalian target of rapamycin
**TFEB**	Transcription factor EB
**ER**	Endoplasmic Reticulum
**ULK**	Unc-51 like autophagy activating kinase
**Unc-51**	Serine/threonine-protein kinase
**RNAi**	Ribonucleic Acid interference
**LPS**	Lipopolysaccharide
**PTEN**	Phosphatase and TENsin homolog deleted on chromosome 10
**IkB**	Inhibitor of kappa-beta
**BCG**	Bacille Calmette-Guerin
**TIR**	Toll/interleukin-1 receptor-like proteindILI
**PAMP**	Pathogen‐associated molecular pattern molecules
**MHC**	Major Histocompatibility Complex
**COX2**	cyclooxygenase-2
**iNOS**	Inducible nitric oxide synthase
**(SHIP1)**	Src homology 2 (SH2) domain containing inositol polyphosphate 5-phosphatase 1
**SOD**	Superoxide dismutase
**APAP**	Acetaminophen
**T-AOC**	Total antioxidant capacity
**AMPK**	Adenosine monophosphate activated protein kinase
**EGF**	Epidermal growth factor
**Fas**	Member of subgroup of a member of a subgroup of the tumour necrosis factor receptor superfamily
**FasR**	Fas-receptor
